# Distinct Roles of Urinary Liver-Type Fatty Acid-Binding Protein in Non-Diabetic Patients with Anemia

**DOI:** 10.1371/journal.pone.0126990

**Published:** 2015-05-26

**Authors:** Naohiko Imai, Takashi Yasuda, Atsuko Kamijo-Ikemori, Yugo Shibagaki, Kenjiro Kimura

**Affiliations:** 1 Division of Nephrology and Hypertension, Department of Internal Medicine, St. Marianna University School of Medicine, Kanagawa, Japan; 2 Department of Internal Medicine, Tokyo Takanawa Hospital, Tokyo, Japan; University of Florida, UNITED STATES

## Abstract

**Background:**

Various stresses including ischemia are known to up-regulate renal L-FABP gene expression and increase the urinary excretion of L-FABP. In diabetic patients with anemia, the urinary excretion of L-FABP is significantly increased. We studied the clinical significance of urinary L-FABP and its relationship with anemia in non-diabetic patients.

**Subjects and Methods:**

A total of 156 patients were studied in this retrospective cross-sectional analysis. The associations between anemia and urinary L-FABP levels, and the predictors of urinary L-FABP levels in non-diabetic patients were evaluated.

**Results:**

Urinary L-FABP levels were significantly higher in patients with anemia compared to those in patients without anemia. Similarly, the urinary L-FABP levels were significantly higher in patients with albuminuria compared to those in patients without albuminuria. Urinary L-FABP levels correlated with urinary albumin-to-creatinine ratios, estimated glomerular filtration rates, body mass index, and hemoglobin levels. Multivariate linear regression analysis determined that hemoglobin levels (β = -0.249, P = 0.001) and urinary albumin-to-creatinine ratios (β = 0.349, P < 0.001) were significant predictors of urinary L-FABP levels.

**Conclusions:**

Urinary L-FABP is strongly associated with anemia in non-diabetic patients.

## Introduction

Liver-type fatty acid-binding protein (L-FABP) is a 14 kDa small molecule that is expressed in the proximal tubular cells of the kidney [1]. L-FABP incorporates albumin-bound free fatty acids (FFAs) that are filtered through the glomeruli into proximal tubular cells, and transports these FFAs from the cytosol to the nucleus [2,3]. Various stresses, such as proteinuria, hyperglycemia, hypertension, ischemia, and toxins are known to up-regulate renal L-FABP gene expression and increase the urinary excretion of L-FABP [4–7].

Anemia has a profound effect on patient’s mortality, morbidity, and quality of life. It also induces tubular hypoxia [8–10]. Administration of erythropoietin and the subsequent increase in hemoglobin levels decreases urinary L-FABP levels [11]. To date, the association between urinary L-FABP and anemia has only been reported among patients with type 2 diabetes [12]. The objective of this study was to study the association between urinary L-FABP and anemia among non-diabetic patients. We hypothesized that there also would be an association between urinary L-FABP and anemia among non-diabetic patients. Thus we conducted a cross-sectional study to investigate urinary L-FABP levels in non-diabetic patients.

## Subjects and Methods

### Patients

Between 2007 and 2011, non-diabetic adult patients were consecutively recruited from the outpatient clinic. Patients with history of liver disease, cancer, collagen disease, and hemodialysis were excluded. Patients were also excluded from this study if their medical records contained inadequate amounts of clinical or biochemical information. Ethical approval was obtained from the Institutional Review Board of St. Marianna University Hospital. The study was conducted in accordance with the principles of the Declaration of Helsinki. All patients were provided written informed consent following confirmation of eligibility.

### Measurements

This was a retrospective cross-sectional study. Patients’ demographic characteristics and laboratory test results were extracted from the electronic patient records and medical notes, including age, sex, body mass index (BMI), hemoglobin (Hb) levels, serum albumin levels, high-sensitive CRP, urinary albumin levels, urinary L-FABP levels, and estimated glomerular filtration rates (eGFR). When immediate analysis is not possible, serum and urine samples were stored at −80°C. Urinary albumin levels were measured using the latex agglutination method. Urinary L-FABP levels were measured using the Human L-FABP ELISA Kit developed by CMIC Co., Ltd. (Tokyo, Japan) [13,14]. Their concentrations were normalized for urine creatinine concentrations. The new equation proposed by the Japanese Society of Nephrology was used to calculate the eGFRs, as follows: eGFR = 194 × (creatinine) ^− 1.094^ × age ^− 0.287^ (or × 0.739 if female) [15]. Anemia was defined using the World Health Organization’s criteria: Hb <13 mg/dL for men and Hb <12 mg/dL for women [16].

### Statistical analysis

Descriptive statistics were used to summarize the demographic characteristics of the patients. For parameters between the two groups, parametric data were compared using unpaired t-tests and non-parametric data were compared using the Mann–Whitney U test. The associations between urinary L-FABP and other variables were evaluated using Spearman’s correlation coefficient. Multivariate linear regression analysis was performed to determine the variables independently predict urinary L-FABP levels. Statistical analyses were performed using IBM SPSS software version 21.0 (IBM Corporation, Armonk, NY, USA). P values <0.05 were considered statistically significant for all analyses.

## Results

A total of 156 non-diabetic patients were studied and their demographic characteristics are summarized (Table 1). The mean age (standard deviation) of the patients was 62.2 years (14.8). The mean eGFR of the patients was 56.6 mL/min/1.73m^2^ (25.0). The median urinary ACR (interquartile range) was 26.4 mg/gCr (7.2–212.3), and the median urinary L-FABP level was 4.5 μg/gCr (0.7–10.2).

**Table 1:** Baseline patient characteristics.

	All patients (n = 156)
Age (years)	62.2 ± 14.8
Female, n (%)	67 (42.9)
Body mass index (kg/m^2^)	24.0 ± 3.6
Systolic blood pressure (mmHg)	130.6 ± 14.5
Diastolic blood pressure (mmHg)	79.1 ± 10.0
eGFR (mL/min/1.73m^2^)	56.6 ± 25.0
Total cholesterol (mg/dL)	183.7 ± 34.7
HDL-cholesterol (mg/dL)	49.5 ± 15.5
High-sensitive CRP (mg/dL)	0.09 (0.05–0.16)
ACE/ARB, n (%)	98 (62.8)
Statin, n (%)	26 (16.7)
Urinary ACR (mg/gCr)	26.4 (7.2–212.3)
Urinary L-FABP (μg/gCr)	4.5 (0.7–10.2)

Data are mean (SD), median (IQR), or number of patients (%). ACR, albumin-to-creatinine ratio; ACE/ARB, angiotensin-converting enzyme/angiotensin-receptor blocker; HDL, high-density lipoprotein; eGFR, estimated glomerular filtration rate.

**Fig 1:**
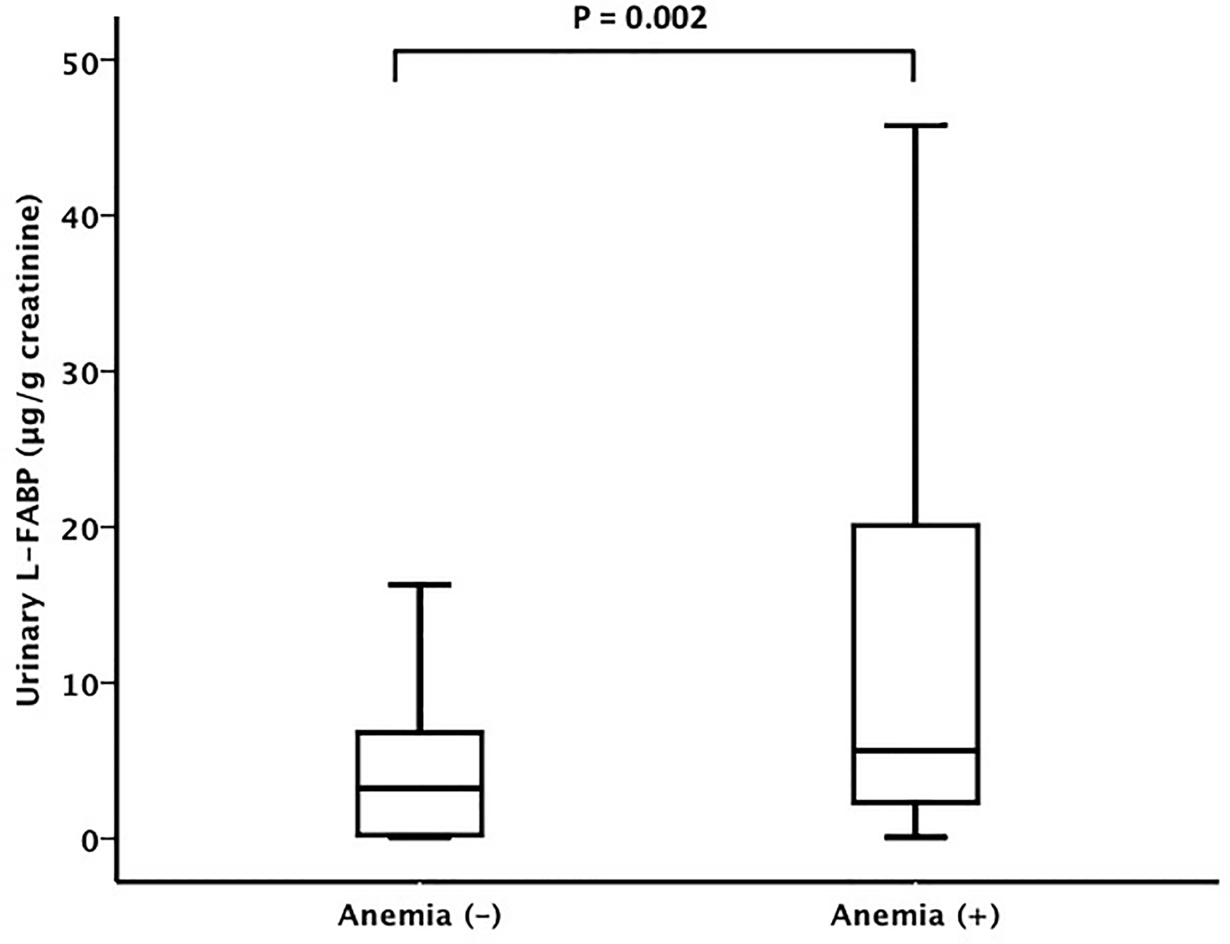
Urinary L-FABP levels and anemia. Patients with anemia had significantly higher urinary L-FABP levels than patients without anemia (5.6 μg/gCr [2.3–20.2] vs. 3.3 μg/gCr [0.2–7.4], P = 0.002).

**Fig 2:**
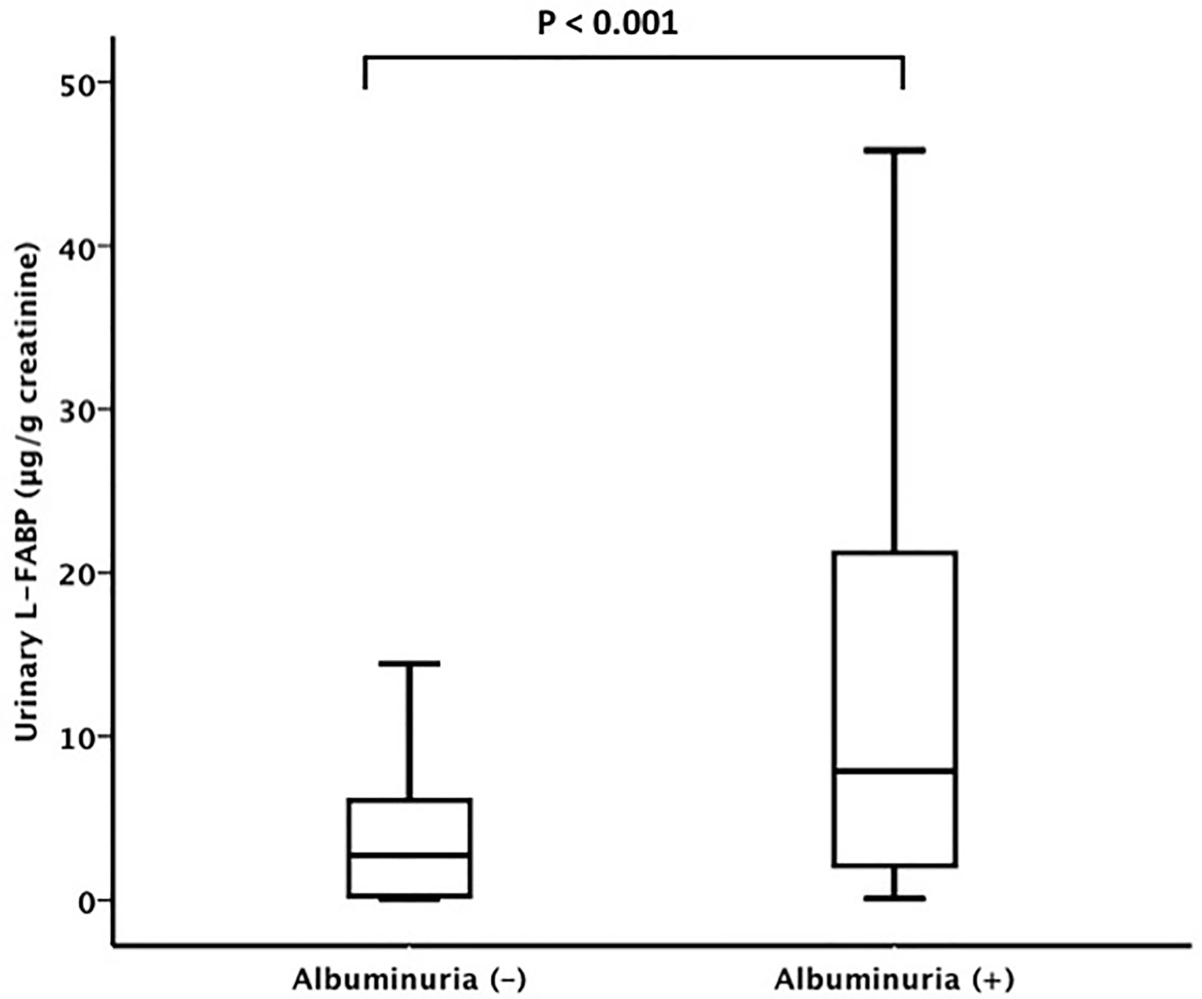
Urinary L-FABP levels and albuminuria. Patients with albuminuria had significantly higher urinary L-FABP levels than patients without albuminuria (7.9 μg/gCr [2.0–21.2] vs. 2.8 μg/gCr [0.3–6.1], P < 0.001).

Patients with anemia had significantly higher urinary L-FABP concentrations compared with patients without anemia (5.6 μg/gCr [2.3–20.2] vs. 3.3 μg/gCr [0.2–7.4], P = 0.002) (Fig 1). Also, patients with albuminuria had significantly higher urinary L-FABP levels than patients without albuminuria (7.9 μg/gCr [2.0–21.2]) vs. 2.8 μg/gCr [0.3–6.1], P < 0.001) (Fig 2).

Urinary L-FABP levels correlated with ACR (r = 0.410, P < 0.001), eGFR (r = -0.364, P < 0.001), BMI (r = -0.277, P = 0.001), Hb levels (r = -0.293, P < 0.001), and the presence of anemia (r = 0.250, P = 0.002) (Table 2). Linear regression analysis, using the urinary L-FABP levels as dependent variables, revealed that Hb (β = -0.249, P = 0.001) and ACR (β = 0.349, P < 0.001) were significant and independent predictors of urinary L-FABP levels (Table 3).

**Table 2:** Spearman’s correlation coefficients between urinary L-FABP levels with clinical characteristic of patients.

	r	P
Age	0.134	0.096
Female sex	-0.062	0.439
Body mass index	-0.277	0.001
Hemoglobin	-0.293	<0.001
Prevalent anemia	0.250	0.002
Total cholesterol	-0.145	0.071
High-sensitive CRP	-0.097	0.228
eGFR	-0.364	<0.001
Urinary ACR	0.410	<0.001

ACR, albumin-to-creatinine ratio; eGFR, estimated glomerular filtration rate.

**Table 3:** Independent predictors of urinary L-FABP^*^ in multivariate linear regression models.

	β	t	P
Age	-0.131	-1.477	0.142
Sex	-0.146	-1.929	0.056
Body mass index	-0.141	-1.690	0.093
Hemoglobin	-0.249	-3.377	0.001
Urinary ACR*	0.349	4.728	<0.001
ACE/ARB	0.128	1.748	0.083
Statin	0.039	0.479	0.632
r^2^			0.197

^*^Log-transformed variables.

ACR, albumin-to-creatinine ratio; eGFR, estimated glomerular filtration rate; ACE/ARB, angiotensin-converting enzyme/angiotensin-receptor blocker.

## Discussion

In this cross-sectional study, we showed for the first time that urinary L-FABP levels are significantly increased in non-diabetic patients with anemia. Similar findings have been reported in experimental models of acute ischemic injury and in diabetic patients [5,12,17]. In the present study, the urinary L-FABP levels were significantly higher (approximately 2-fold) in patients with anemia compared to those in patients without anemia. Urinary L-FABP levels were similar to previously reported levels in diabetic patients with anemia [12]. Also, patients with albuminuria had urinary L-FABP levels that were significantly higher (approximately 3-fold) compared to those in patients without albuminuria. Multivariate linear regression analysis identified Hb levels and the ACR as significant predictors of urinary L-FABP levels. These results are similar to those reported in the study of diabetic patients, which reported that Hb levels and the ACR were significant predictors of urinary L-FABP levels [12].

Serum LFABP levels do not affect urinary L-FABP levels. A previous study has reported that the estimated contribution of serum L-FABP to urinary L-FABP is only 3% [18]. This suggests that there is no transglomerular passage of L-FABP, and that it is the tubular cells that primarily produce urinary L-FABP. It is shown that administration of erythropoietin and the subsequent increase in hemoglobin levels decreases urinary L-FABP levels [11]. Tubular hypoxia induced by anemia likely up-regulate expression of the LFABP gene and promote the urinary excretion of LFABP [5]. On the other hand, albumin is transported with FFAs to the proximal tubules, where the tubular cells absorb the FFAs. Subsequently, L-FABP transports the FFAs to the mitochondria. Hence, when the severity of albuminuria increases, the L-FABP gene is up-regulated, and more LFABP is excreted in the urine [19,20].

In the present study, statins and angiotensin receptor blockers (ARBs) were administered to 17% (n = 26) and 63% (n = 98) of patients, respectively. Statin use has been shown to decrease proliferation, increase apoptosis, and enhance the fibrinolytic activity of renal tubular cells, while ARB use has been shown to prevent vascular damage, ameliorate tubular hypoxia, and reduce oxidative stress [21,22]. Previous studies have reported a significant decrease in urinary L-FABP when the patients were treated with statins or ARBs [19,23,24]. Therefore, statins and/or ARBs might have influenced the changes in the urinary L-FABP levels observed among our patients. Other reports have also suggested that angiotensin-converting enzyme inhibitors (ACEi) and ARBs might have a negative impact on Hb levels [25,26]. Although a high proportion of our patients (66%, n = 103) were receiving these drugs, their use did not appear to be independently associated with lower Hb levels in our patients, as has been previously reported [27,28].

Our study has several limitations. First, this was a single-center study and the sample size was relatively small. Second, as in every cross-sectional study, no clear conclusions can be reached regarding the associations between the parameters studied. As well, bias by indication is also possible. Third, erythropoietin and iron levels were not measured for any of the subjects. Since patients are more elderly and more male subjects are included, an iron deficiency might not be the reason for anemia in some of the patients. Fourthly, we were not able to compare urinary L-FABP with other emerging markers of kidney dysfunction such as kidney injury molecule (KIM) -1, N-acetyl-β-glucosaminidase (NAG), and neutrophil gelatinase-associated lipocalin (NGAL). Further study is needed to evaluate the clinical value of urinary L-FABP. Finally, there are no marker of hypoxia, such as lactate and pH, available in the present study. Anemic patients with high urinary L-FABP levels may benefit from therapeutic interventions that address renal tubular hypoxia. This also would be the area where further research is needed.

Based on the findings of this study, urinary L-FABP levels have a strong association with anemia. Further prospective studies are needed to clarify the utility of measuring urinary L-FABP levels in anemic patients.
